# Predictive Value of Stemness Factor Sox2 in Gastric Cancer Is Associated with Tumor Location and Stage

**DOI:** 10.1371/journal.pone.0169124

**Published:** 2017-01-03

**Authors:** Lang Yang, Jun-Feng Xu, Qian Kang, Ai-Qin Li, Peng Jin, Xin Wang, Yu-Qi He, Na Li, Tao Cheng, Jian-Qiu Sheng

**Affiliations:** 1 Department of Gastroenterology, PLA Army General Hospital, Beijing, China; 2 GraduateCollege, PLA General Hospital, Beijing, China; 3 Department of Pathology, PLA Army General Hospital, Beijing, China; National Cancer Center, JAPAN

## Abstract

Cancer stem cells (CSCs) are thought to be the "root" of cancer. Although stemness-related factors ALDH1A1 and Sox2 have been used as markers to identify gastric CSCs, the expression pattern and significance of these factors in gastric cancer have not been sufficiently demonstrated. In this study, the expressions of ALDH1A1 and Sox2 were detected by immunohistochemistry in 122 gastric cancer specimens. And the correlation between Sox2 or ALDH1A1 expression and clinicopathological parameters and overall survival data were analyzed. The positive rate of ALDH1A1 expression was 60%, but there was no significant difference between survival rates of ALDH1A1-positive and ALDH1A1-negative patients. Sox2 was expressed in 42% of specimens and was associated with poor prognosis of patients (*P* = 0.015). Stratified analysis showed that Sox2 expression correlated with shorter lifespan only in patients with cardiac gastric cancers (*P* = 0.002) or stage I or II gastric cancers (*P* = 0.002); but not in patients with non-cardiac cancers (*P* = 0.556) or stage III or IV gastric cancers (*P = 0*.*121*). Analysis on a database cohort validated the correlation between Sox2 expression and poor prognosis in stage II cancer. Also, expression of Sox2 was associated with lymphnode metastasis in patients with cardiac gastric cancer (*P* = 0.037). A multivariate analysis revealed that Sox2 was an independent prognostic factor in cardiac gastric cancer. Our results indicate that predictive value of Sox2 in gastric cancer is associated with cardiac cancer location and with early cancer stages (I and II).

## Introduction

Gastric cancer is one of the most common malignancies worldwide, especially in Eastern Asia, Central and Eastern Europe, and South America[1]. Complete resection of the tumor and adjacent lymphnodes is the only effective curative treatment. Despite the tremendous improvements in surgery and chemotherapy, the five-year survival rate remains low owing to the nature of metastasis and recurrence [2–4].

Recently, the cancer stem cell (CSC) theory has become a highlight of the cancer research field. CSCs have been identified not only in leukemia, but also in solid tumors, including gastric cancer [5–6]. CSCs play important roles in tumor progression and recurrence[7], and provide a potential therapeutic target[8]. In gastric cancer, stem cell related factors ALDH1 and Sox2 were used as markers to identify the gastric CSCs[6, 9–10]. However, the relationship between these factors and patient prognosis remains to be illustrated. Some reports showed that Sox2 expression was associated with poorer overall survival in gastric cancer[11–12]; however, others argued that the expression of Sox2 decreased during gastric carcinogenesis and this was predictive of better survival[13]. However, no significant correlation between Sox2 and survival has been presented[14]. In addition, the predicted prognostic value of ALDH1A1 in gastric cancer remained inconsistent; ALDH1A1 was found to be associated with a poor prognosis of gastric cancer[15], while Wakamatsu et al. demonstrated that ALDH1A1 expression, whether high or low, showed no correlation with survival[16]. Therefore, the role of Sox2 and ALDH1A1 in gastric cancer remains nebulous.

Gastric cancer is a heterogeneous disease that is often reported as a single entity. Topographically, gastric cancer can be classified into cardiac gastric cancer and non-cardiac gastric cancer[17]. In this study, we hypothesized that inconsistency in the published results may be related to stratified pathological factors. Therefore, we evaluated the expression of ALDH1A1 and Sox2 in gastric cancer samples, and, with respect to stratification, analyzed their correlation with pathological parameters and patient survival.

## Materials and Methods

### Patients and specimens

A total of 122 Gastric adenocarcinoma tissues were collected from 2010 to 2013 at PLA Army General Hospital (Beijing, China). No preoperative radiotherapy or chemotherapy was performed before surgery for the patients; patients were monitored every three to six months. There were 100 male patients and 22 female patients. The mean age is 63 years (range, 29–82 years). Tumor stage was classified according to the 7th Union International Cancer Control (UICC) TNM staging system. To analyze for stratification based on the tumor location, gastric cancers that meet Siewert type II and III, according to Siewert classification[18], were classified as cardiac gastric cancer, while the rest were classified as non-cardiac cancer. All tissue samples were fixed in 10% neutral formalin and embedded in paraffin. A tissue array was constructed and cut into 4-μm sections. This study was approved by the ethical review committee of PLA Army General Hospital; written informed consent was obtained from each patient.

### Immunohistochemistry

Immunohistochemistry was performed as described[19]. Briefly, the tissue array section was dewaxed with xylene, rehydrated, pretreated with 3% H_2_O_2_, and then was subjected to antigen retrieval. The slides were incubated with mouse anti-human ALDH1A1 (dilution 1:1000; BD Bioscience, USA) or rabbit anti-human Sox2 (dilution 1:200; Cell Signaling Technology, USA) at 4 Covernight. The horseradish peroxidase (HRP) labeled secondary antibody was added for 30 minutes at room temperature. At last, Reactions were revealed with 3, 3′-diaminobenzidine (DAB, Zhongshan Gold Bridge Corporation, China). Slides were then counterstained with hematoxylin, dehydrated with alcohol and xylene, and mounted on cover slips. The Immunohistochemistry results were interpreted as follows [11, 19–20]: briefly, the cells with brown color in the cytoplasm for ALDH1A1 and nuclei for Sox2 staining were counted as positive cells. At least five fields were randomly selected for calculating average percentage of positive cells over total cancer cells. The sections with less than 5% positive cancer cells were designated as negative and sections with more than 5% positive cancer cells were designated as positive.

### Statistical analysis

All data were analyzed with SPSS13.0 statistical software (SPSS Inc. Chicago, IL). The Pearson Chi-square test and Fisher’s exact test were used to evaluate comparisons of clinicopathological characteristics. Kaplan–Meier survival plots and log-rank statistics were used to compare the survival rates of patients with follow-up data. Multivariate analyses were performed using the Cox proportional hazards regression model. Variables with *P* value < 0.05 in univariate analysis were used in the multivariate regression. *P* values<0.05 were considered statistically significant.

## Results

### Expression of Sox2 and ALDH1A1 in gastric cancer

**Fig 1:**
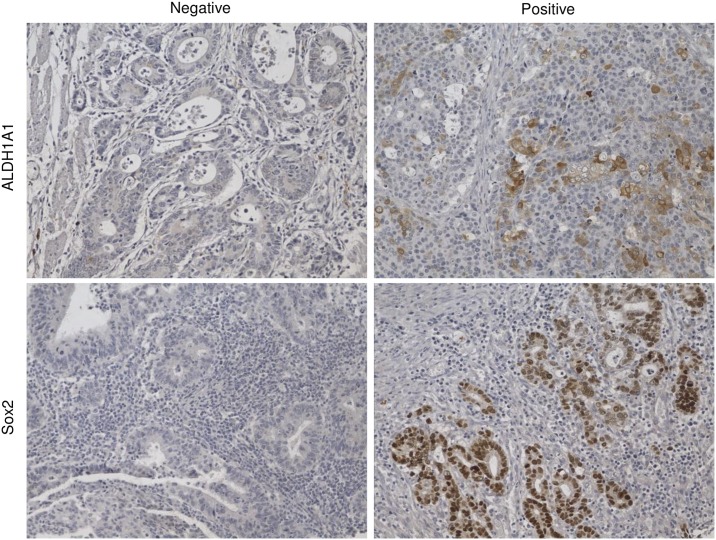
Representative picture of Sox2 and ALDH1A1 expression in gastric cancer.

Sox2 was found located in nuclei and ALDH1A1 was stained in cell plasma (Fig 1). Forty-two percent (51/122) of gastric cancer samples showed Sox2 positive expression (Table 1). No correlation was found between Sox2 expression and age (*P* = 0.375), sex(*P* = 0.701), Lauren classification(*P* = 0.623), cardiac cancer(*P* = 0.199), T stage(*P* = 0.430), N stage(*P* = 0.166), TNM stage (*P* = 0.894) or ALDH1A1 expression(*P* = 0.192) in gastric cancer (Table 1). Sixty percent (73/122) of gastric cancer samples showed ALDH1A1 positive expression (S1 Table). The expression of ALDH1A1 was positively correlated with cardiac cancer (*P* = 0.006). No correlation was found between ALDH1A1 expression in gastric cancer and age (*P* = 0.194), sex(*P* = 0.688), Lauren classification(*P* = 0.856), T stage(*P* = 0.050), N stage(*P* = 0.390), or TNM stage(*P* = 0.118) (S1 Table).

**Table 1:** The Relationship between Sox2 and Clinicopathological Parameters in Total Gastric Cancer.

Parameter	Total gastric cancer			P value
	Total	Sox2 +	Sox2 -	
*Age (years)*				
≤60	56	21	35	0.375
>60	66	30	36	
*Sex*				
male	100	41	59	0.701
female	22	10	12	
*Lauren classification*				
intestinal	71	31	40	0.623
diffuse	51	20	31	
*Location*				
cardiac	61	29	32	0.199
non-cardiac	61	22	39	
*Invasive depth*				
T1+T2	18	6	12	0.430
T3+ T4	104	45	59	
*Lymph node metastasis*				
presence	85	39	46	0.166
absence	37	12	25	
*TNM stage*				
I+ II	47	20	27	0.894
III+ IV	75	31	44	
*ALDH1A1 expression*				
negative	49	17	32	0.192
positive	73	34	39	

### Correlations between Sox2 and ALDH1A1 expression and patient survival time

**Fig 2:**
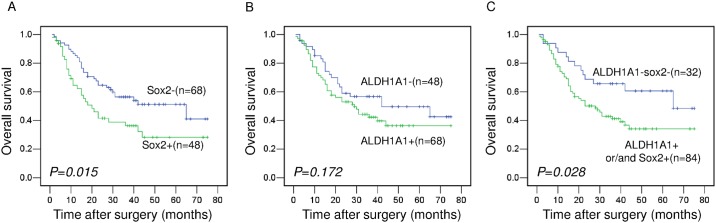
Kaplan–Meier survival curves of patients with gastric cancer according to the expression of Sox2 and ALDH1A1. (A) Patients with Sox2 positive gastric cancers have poor survival for all patients; (B) Patients has similar survival rates regardless of ALDH1A1 expression. (C) Combined negative expression of ALDH1A1 and Sox2 predict better survival in patients with gastric cancer.

We analyzed how the expression levels of Sox2 and ALDH1A1 individually correlate with overall survival in a total 116 gastric cancer patients with follow-up data. Median survival time of the 116 gastric cancer patients was 26 months (ranging from 1 to 75 months) and Fifty-three percent (62/116) were deceased at this time point. Kaplan–Meier survival curves showed that patients with Sox2 negative tumors had better prognoses compared to those with Sox2 positive tumors (Fig 2A, *P* = 0.015). In addition, similar survival rates were observed regardless of ALDH1A1 positivity (Fig 2B, *P* = 0.172) while patients with negative ALDH1A1 and Sox2 expression has higher survival rate than patients with ALDH1A1 and/or Sox2 positive expression (Fig 2C, *P* = 0.028 and S1 Fig). In addition, we also use the database (http://kmplot.com/analysis/) to analyze the relationship between ALDH1A1 and Sox2 mRNA levels and survival of gastric cancer patients; we found that high ALDH1A1 mRNA levels were associated with better survival, while, high Sox2 mRNA levels were associated with poor prognosis (S2 Fig). Moreover, a multivariate analysis revealed that the expression of Sox2, TNM stage and diffuse type histology were independent prognostic factors for overall survival (Table 2).

**Table 2:** Univariate and Multivariate Cox Models for the Association between Survival and Clinicopathological Factors in Patients with Gastric Cancer.

Variables	Univariate Analysis		Multivariate analysis	
	Hazard ratio (95% CI)	P value	Hazard ratio (95% CI)	P value
Total gastric cancer				
Age (≤60 vs. >60 years)	1.54(0.93–2.56)	0.096		
Sex (male vs. female)	1.13(0.59–2.17)	0.712		
Location (cardiac vs. non-cardiac)	0.93(0.56–1.53)	0.772		
Lauren classification (intestinal vs. diffuse)	2.52(1.51–4.20)	**0.000**	2.00(1.17–3.40)	**0.011**
Invasive depth (T1, T2 vs. T3, T4)	4.83(1.51–15.43)	**0.008**	1.81(0.53–6.21)	0.345
Lymph node metastasis (N0 vs. N1-N3)	4.88(2.31–10.30)	**0.000**	1.49(0.50–4.41)	0.476
TNM stage (I, II vs.III, IV)	4.66(2.45–8.86)	**0.000**	3.15(1.20–8.31)	**0.020**
ALDH1A1(negative vs. positive)	1.43(0.85–2.40)	0.179		
Sox2(negative vs. positive)	1.83(1.11–3.02)	**0.017**	2.02(1.20–3.40)	**0.008**
Cardiac gastric cancer				
Age (≤60 vs. >60 years)	1.48(0.65–3.34)	0.350		
Sex (male vs. female)	1.66(0.62–4.45)	0.316		
Lauren classification (intestinal vs. diffuse)	4.36(1.98–9.56)	**0.000**	2.93(1.29–6.67)	**0.011**
Invasive depth (T1, T2 vs. T3, T4)	2.32(0.55–9.81)	0.253		
Lymph node metastasis (N0 vs. N1-N3)	6.76(2.34–19.57)	**0.000**	1.72(0.35–8.34)	0.502
TNM stage (I, II vs.III, IV)	5.47(2.18–13.69)	**0.000**	2.57(0.71–9.39)	0.152
ALDH1A1(negative vs. positive)	1.20(0.51–2.83)	0.676		
Sox2(negative vs. positive)	3.28(1.50–7.15)	**0.003**	2.44(1.09–5.50)	**0.031**

### Predictive value of ALDH1A1 and Sox2 with respect to tumor stage and location

**Fig 3:**
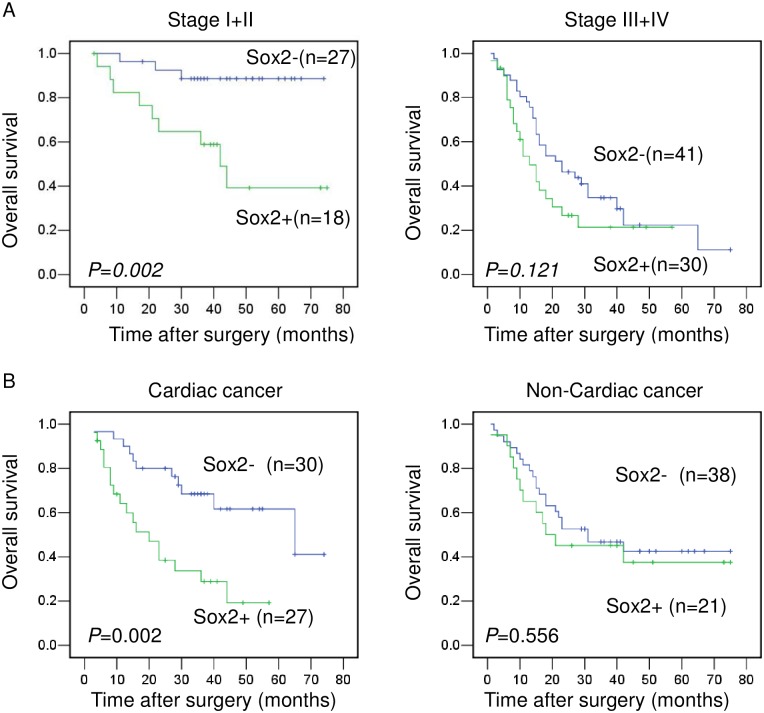
Kaplan-Meier survival curves of patients with different stages and tumor locations according to Sox2 expression. **(A)** Overall survival of patients in the Stage I and II group and in the Stage III and IV group. (B) Overall survival of patients in the cardiac gastric cancer and in non-cardiac cancer groups.

**Fig 4:**
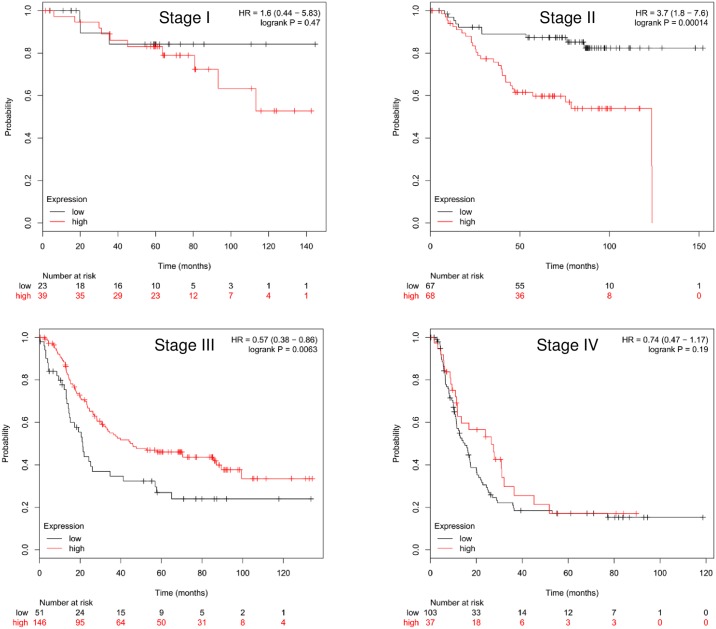
Kaplan-Meier survival curves of patients with different stage associated with to Sox2 mRNA level (228038_at).

We also analyzed, with respect to stratification, the predictive value of Sox2 and ALDH1A1 expression in relation to stage and location. We found that Sox2 positive expression was associated with shorter survival in overall patients at Stages I and II, but not at Stages III and IV (Fig 3A and S3 Fig). Univariate analysis showed that expression of Sox2 was the only factor associated with prognosis for overall patients with gastric cancer at Stages I and II (Table 3). It is of interest that high Sox2 mRNA levels were associated with poor prognosis in Stage II, but with better survival in Stage III, based on the data from http://kmplot.com/analysis/ (Fig 4).

**Table 3:** Univariate Analysis for the Association between Survival and Clinicopathological Factors in Patients with Gastric Cancer at Stages I and II.

Variables	Hazard ratio (95% CI)	*P* value
Stage I and II		
Age (≤60 vs. >60 years)	2.26(0.68–7.51)	0.185
Sex (male vs. female)	0.99(0.13–7.64)	0.985
Location (cardiac vs. non-cardiac)	0.90(0.30–2.81	0.858
Lauren classification (intestinal vs. diffuse)	2.06(0.65–6.52)	0.218
Invasive depth (T1, T2 vs. T3, T4)	2.71(0.59–12.39)	0.198
Lymph node metastasis (N0 vs. N1-N3)	2.56(0.81–8.10)	0.109
TNM stage (I vs. II)	4.23(0.55–32.76)	0.168
ALDH1A1(negative vs. positive)	0.97(0.31–3.02)	0.962
Sox2(negative vs. positive)	6.06(1.63–22.46)	**0.007**

The predictive value of Sox2 expression in patients with cardiac or non-cardiac gastric cancer was analyzed. We found that patients with Sox2 negative cardiac cancers have better survival than those with Sox2 positive cardiac cancers. However, Sox2 expression status had no bearing on survival rates in in non-cardiac gastric cancers (Fig 3B). In addition, the interactions between SOX2 expression and tumor staging or location in relationship to survival were analyzed by using Cox regression model, no interaction was found between Sox2 expression and tumor location or staging in relationship to survival (S2 Table). Multivariate analysis revealed that Sox2 expression and diffuse type histology were independent prognostic factors for overall survival in cardiac gastric cancer (Table 2).

Moreover, we analyzed the relationship between Sox2 and clinical parameters in cardiac gastric cancer, and found that Sox2 expression was positively associate with lymphnode metastasis (Table 4, *P* = 0.037). There were no associations found between Sox2 expression and age, sex, Lauren classification, invasion depth, and TNM stage in cardiac gastric cancers (Table 4),and there were no associations found between Sox2 expression and the same clinicopathological factors in non-cardiac gastric cancer (S3 Table).

**Table 4:** The Relationship between Sox2 and Clinicopathological Parameter in Cardiac Gastric Cancer.

Parameter	Cardiac gastric cancer (N)			P value
	Total	Sox2 +	Sox2 -	
*Age (years)*				
≤60	21	9	12	0.596
>60	40	20	20	
*Sex*				
male	51	25	26	0.735^a^
female	10	4	6	
*Lauren classification*				
intestinal	37	17	20	0.757
diffuse	24	12	12	
*Invasive depth*				
T1+ T2	7	1	6	0.106^a^
T3+ T4	54	28	26	
*Lymph node metastasis*				
presence	38	22	16	**0.037**
absence	23	7	16	
*TNM stage*				
I+ II	26	10	16	0.221
III+ IV	35	19	16	
*ALDH1A1 expression*				
negative	17	7	10	0.536
positive	44	22	22	

^a.^ Fisher’s exact test

ALDH1A1 expression was not associated with patient survival with respect to tumor stage and location, although it seemed that patients with ALDH1A1 negative non-cardiac cancer had better survival rates than those with ALDH1A1 positive non-cardiac cancers, but there was no statistically significant difference (S4 Fig).

## Discussion

Although rapid progress has been made in basic and translational research on CSCs in the past decade, the precise isolation and identification of CSCs by appropriate experimental methods and markers remains a challenge in the field of CSC research[21]. Gastric CSCs can be isolated or enriched by stem cell specific markers, side-population (SP) phenotypes, or characteristics[22–24] that include spherical growth in conditioned medium[10] and resistance to chemotherapy drugs[25]. ALDH1 activity and Sox2 expression measurements were employed as markers for the isolation and characterization of gastric CSCs[9, 26–29]. However, the association of ALDH1 and Sox2 expression with pathological parameters and patient survival in gastric cancer remains controversial.

A meta-analysis revealed that Sox2 over-expression was associated neither with the overall survival nor with the other clinicopathological factors with obvious heterogeneity[30–31]. In our study, we analyzed the data, with respect to stratification, based on tumor location and stage, we found that Sox2 had predictive value in cardiac gastric cancers or earlier stage (Stages I and II), but not in non-cardiac gastric cancers or later stage (Stages III and IV). The results based on the database also supported the hypothesis that Sox2 expression is associated with a poor prognosis at earlier tumor stages. Our data suggested that tumor location and stage might be factors that have resulted in inconsistent published data.

Adenocarcinoma of esophagogastric junction (AEG) can be divided to three types according to Siewert classification[18]. The Siewert type II AEG and the Siewert type III AEG (which we referred to as cardiac gastric cancer in this paper) are recommended to be considered as gastric cancers[32–33]. The incidence of AEG is increasing in the western countries, while there is no obvious evidence that indicates a rapid increase of AEG in the eastern countries[33]; however, the proportion of incidence of cardiac cancers to that of total gastric cancers has sharply increased in China[34]. In addition, gastric cardiac cancers (Siewert type II and III) have distinct clinicopathological features and risk factors that clearly differ from those of non-cardiac gastric cancers[17, 35]. Based on our data, the percentage of cardiac cancers may influence the result of predictive value of Sox2 in total gastric cancer.

Sox2 is a transcription factor that plays an important role in fetal development and in cancer biology[36–37]. Sox2 positive stomach cells possess stem cell properties, and can differentiate into all cell types in both the pylorus and corpus glands[38]. The role of Sox2, however, was shown to be paradoxical in gastric cancer[37]. Sox2 was reported as a tumor suppressor that inhibits cell growth by either regulating cyclin D1, phosphorylated Rb, or p27 (Kip1) levels[39], or directly activating PTEN[13]. In addition, Sox2 has been shown to inhibit migration and invasion by upregulating p21 expression in gastric cancer[40]. However, others demonstrated that Sox2 operates as an oncogene in gastric cancer. Blocking Sox2 has been shown to reduce gastric cancer cell proliferation, migration, and tumorigenic potential[41], and impair the cancer stem cell like phenotype[42]. In our study, we found that Sox2 was associated with lymphnode metastasis in gastric cardiac cancer (Siewert type II and III); however, the exact mechanism by which Sox2 correlated with poor survival in cardiac gastric cancer but not with non-cardiac gastric cancer needs further research.

ALDH1 is a predominant isoform of aldehyde dehydrogenase, which participates in the metabolism of a wide variety of aliphatic and aromatic aldehydes[43–44]. The observation that cancer cells with high ALDH1 actively possess CSC properties was reported in various tumor types including breast cancer[45], esophageal squamous cell cancer[19], colon cancer[46], lung cancer [47], and gastric cancer[9, 26]. However, the expression and significance of ALDH1A1in gastric cancer is still unclear. Shen et al. found that ALDH1A1 mRNA was downregulated in gastric cancer and that high ALDH1A1 mRNA level was associated with better overall survival in gastric cancer patients, and was predictive of better survival in gastric intestinal type cancer, but not in diffuse type cancer[48]. Wakamatsu et al. found ALDH1 positivity to be significantly higher in T stage and TNM stage; however, this result did not correlate with any prognostic impact[16]. Li et al. reported that ALDH1A1 was an independent prognostic factor for both overall survival and recurrence-free survival[15]. In our study, we found that ALDH1A1 positive expression was neither associated with survival data nor with clinicopathological factors except for cardiac cancer location.

This study is limited owing to its small cohort size. Predictive survival in relation to Sox2 expression could not be addressed with respect to each TNM stage.

In conclusion, we found that positive Sox2 expression was associated with worse survival in patients with cardiac gastric cancer and earlier cancer stages (Stages I and II). Tumor location and stage may be important factors involved in gastric cancer heterogeneity, and should be considered in future studies.

## Supporting Information

S1 Fig Kaplan-Meier survival curves of patients with gastric cancer according to the joint expression of Sox2 and ALDH1A1. (TIFF)

S2 Fig The prognostic value of mRNA level of ALDH1A1 and Sox2 in all gastric cancer patients assessed by the Kaplan-Meier plotter. (A) ALDH1A1 (212224_at); (B) Sox2 (228038_at).
(TIFF)

S3 Fig Kaplan-Meier survival curves of patients with stage III gastric cancer according to Sox2 expression. (TIFF)

S4 Fig Kaplan-Meier survival curves of patients with different stages and tumor locations associated with ALDH1A1 expression. **(A)** Overall survival of patients in the Stage I and II group and in the Stage III and IV group. (B) Overall survival of patients in the cardiac gastric cancer and in non-cardiac cancer groups.
(TIFF)

S1 Table The Relationship between ALDH1A1 and Clinicopathological Parameter in Total Gastric Cancer. (DOCX)

S2 Table Cox Regression Model for the Interactions betweenSOX2 Expression and Location or Staging Adjusting for Age and Sex. (DOCX)

S3 Table The Relationship between Sox2 and Clinicopathological Parameter in Non-Cardiac Gastric Cancer. (DOCX)
