# Supplementary figures and images for: Predictive Value of Stemness Factor Sox2 in Gastric Cancer Is Associated with Tumor Location and Stage
Source: PLoS One. 2017 Jan 3;12(1):e0169124. doi: 10.1371/journal.pone.0169124 (PMC5207680; doi:10.1371/journal.pone.0169124)

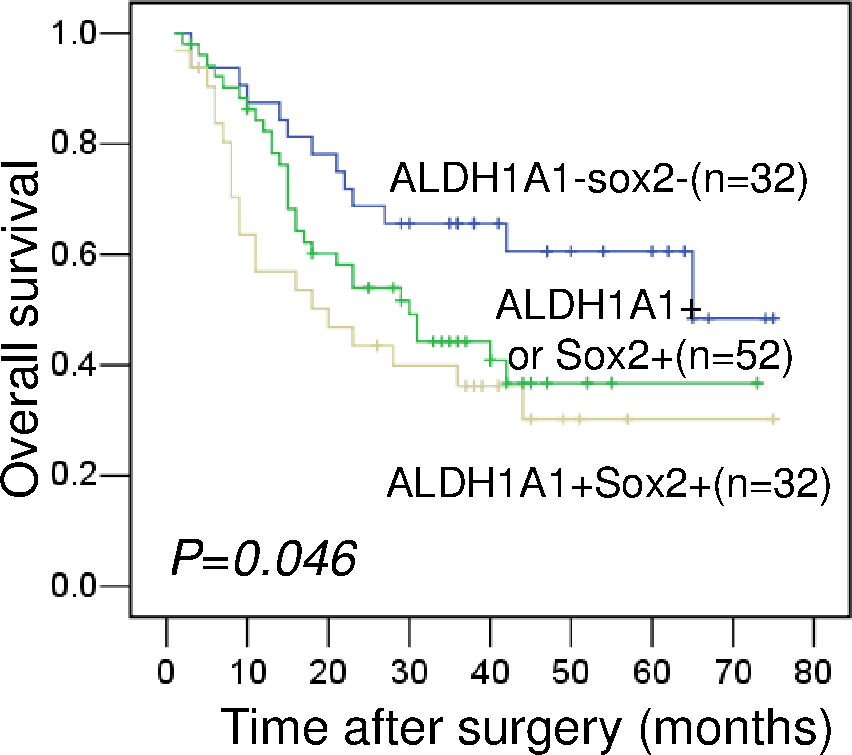

Supplement: S1 Fig — (TIFF) [file pone.0169124.s001.tiff]

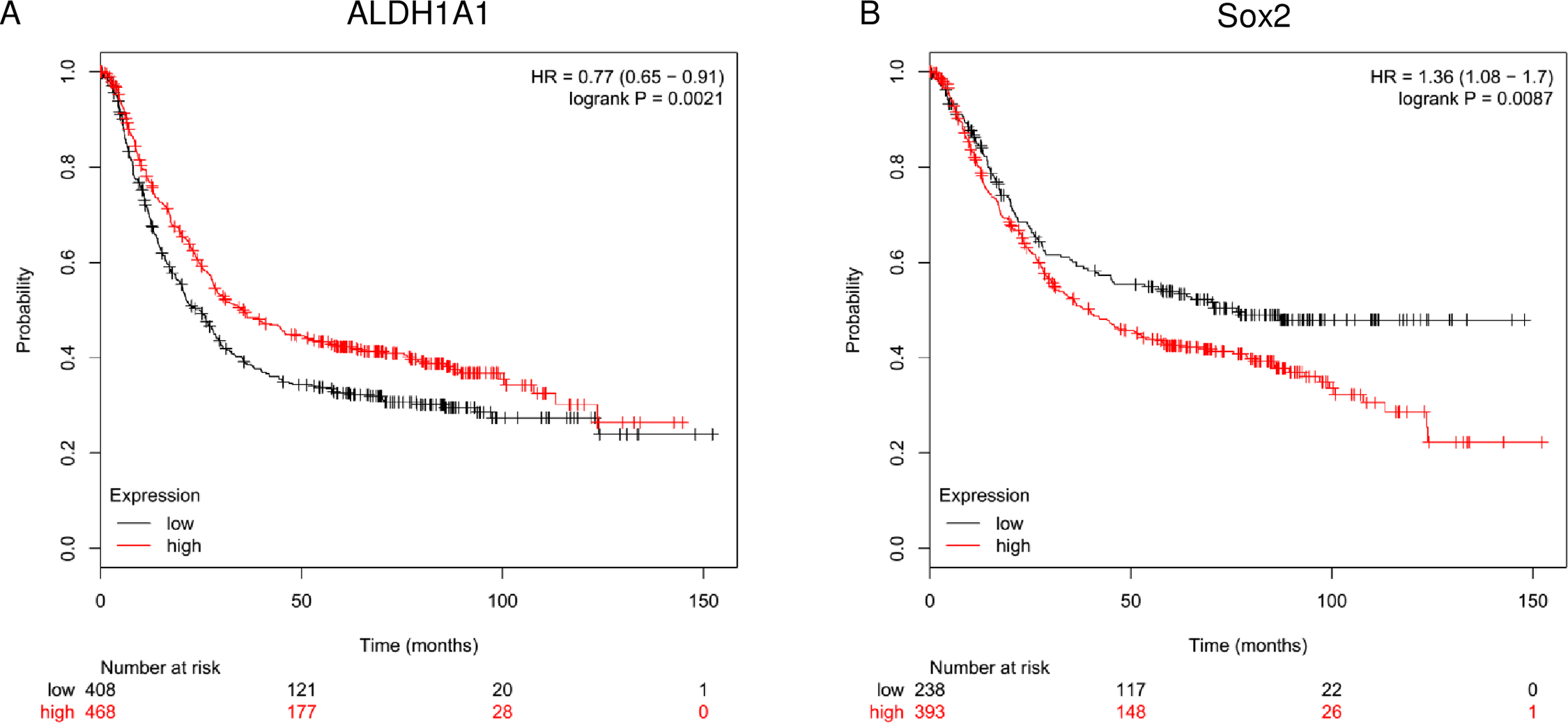

Supplement: S2 Fig — (A) ALDH1A1 (212224_at); (B) Sox2 (228038_at). (TIFF) [file pone.0169124.s002.tiff]

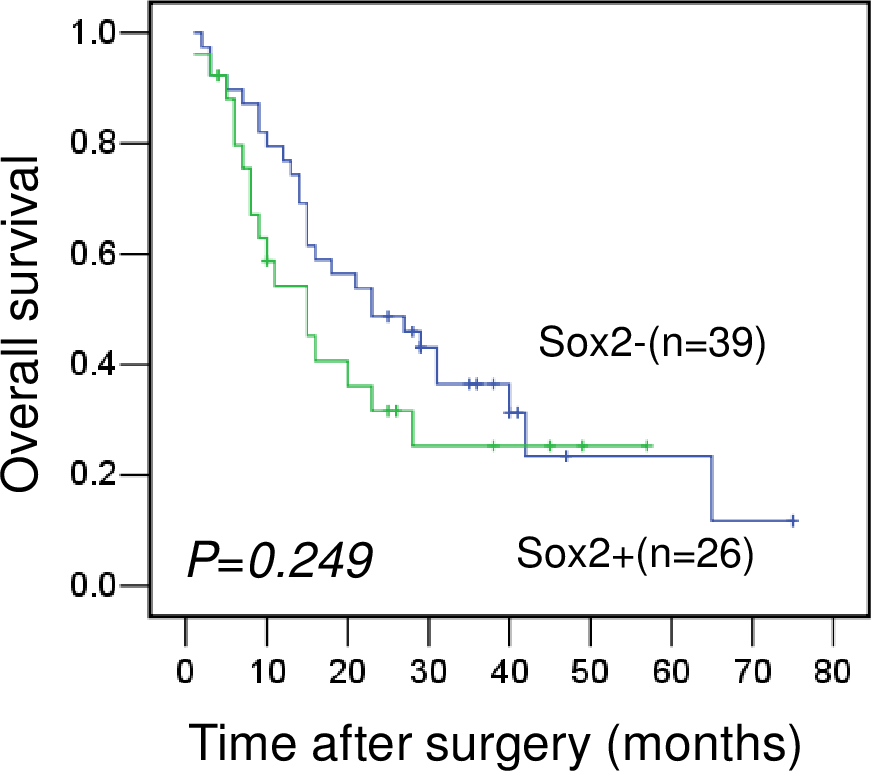

Supplement: S3 Fig — (TIFF) [file pone.0169124.s003.tiff]

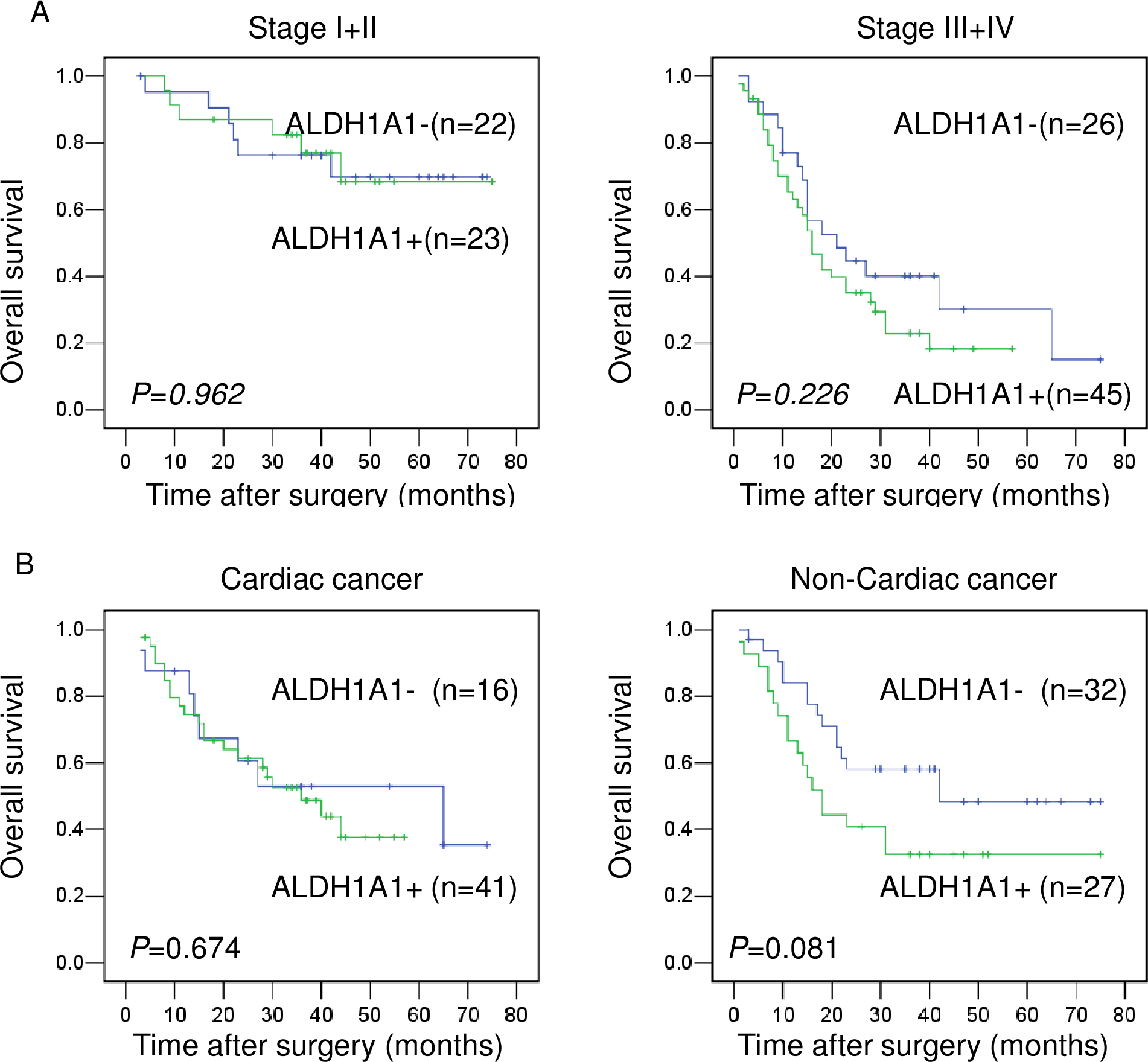

Supplement: S4 Fig — (A) Overall survival of patients in the Stage I and II group and in the Stage III and IV group. (B) Overall survival of patients in the cardiac gastric cancer and in non-cardiac cancer groups. (TIFF) [file pone.0169124.s004.tiff]
