# Supplementary material for: Predictive Value of Stemness Factor Sox2 in Gastric Cancer Is Associated with Tumor Location and Stage
Source: PLoS One. 2017 Jan 3;12(1):e0169124. doi: 10.1371/journal.pone.0169124 (PMC5207680; doi:10.1371/journal.pone.0169124)
Supplement: S1 Table — (DOCX) [file pone.0169124.s005.docx]

**S1 Table The Relationship between ALDH1A1 and Clinicopathological Parameter in Total Gastric Cancer**

| Parameter | Total gastric cancer | | | P value |
| --- | --- | --- | --- | --- |
|  | Total | ALDH1A1 + | ALDH1A1 - |  |
| *Age* | | | | |
| <=60 | 56 | 30 | 26 | 0.194 |
| >60 | 66 | 43 | 23 |  |
| *Gender* | | | | |
| male | 100 | 59 | 41 | 0.688 |
| female | 22 | 14 | 8 |  |
| *Lauren classification* | | | | |
| intestinal | 71 | 42 | 29 | 0.856 |
| diffuse | 51 | 31 | 20 |  |
| *Location* | | | | |
| cardiac | 61 | 44 | 17 | 0.006 |
| No-cardiac | 61 | 29 | 32 |  |
| *Invasive depth* | | | | |
| T1+T2 | 18 | 7 | 11 | 0.050 |
| T3+ T4 | 104 | 66 | 38 |  |
| *Lymph node metastasis* | | | | |
| presence | 85 | 53 | 32 | 0.390 |
| absence | 37 | 20 | 17 |  |
| *TNM stage* |  |  |  |  |
| I+II | 47 | 24 | 23 | 0.118 |
| III+ IV | 75 | 49 | 26 |  |
