# Supplementary material for: Predictive Value of Stemness Factor Sox2 in Gastric Cancer Is Associated with Tumor Location and Stage
Source: PLoS One. 2017 Jan 3;12(1):e0169124. doi: 10.1371/journal.pone.0169124 (PMC5207680; doi:10.1371/journal.pone.0169124)
Supplement: S2 Table — (DOCX) [file pone.0169124.s006.docx]

Table S2. Cox Regression Model for the Interactions between SOX2 Expression and Location or Staging Adjusting for Age and Sex

| Variables | Hazard ratio (95% CI) | P value |
| --- | --- | --- |
| Age (≤60 vs. >60) | 1.53（0.90-2.60） | 0.118 |
| Sex (male vs. female) | 0.82（0.43-1.60） | 0.566 |
| Location (cardiac vs. non-cardiac) | 0.78（0.36-1.66） | 0.513 |
| TNM stage (I, II vs. III, IV) | 10.33（3.06-34.87） | **0.000** |
| Sox2( negative vs. positive) | 18.56（1.17-295.05） | **0.038** |
| Sox2 × TNM stage | 0.26（0.06-1.13） | 0.073 |
| Sox2 × Location | 1.37（0.48-3.91） | 0.563 |
