# Supplementary material for: Predictive Value of Stemness Factor Sox2 in Gastric Cancer Is Associated with Tumor Location and Stage
Source: PLoS One. 2017 Jan 3;12(1):e0169124. doi: 10.1371/journal.pone.0169124 (PMC5207680; doi:10.1371/journal.pone.0169124)
Supplement: S3 Table — (DOCX) [file pone.0169124.s007.docx]

**S3 Table the relationship between Sox2 and clinicopathological parameter in Non-Cardiac gastric cancer**

| Parameter | Non-cardiac gastric cancer | | | P value |
| --- | --- | --- | --- | --- |
|  | Total | Sox2 + | Sox2 - |  |
| *Age* | | | | |
| <=60 | 35 | 12 | 23 | 0.737 |
| >60 | 26 | 10 | 16 |  |
| *Gender* | | | | |
| male | 49 | 16 | 33 | 0.322^a^ |
| female | 12 | 6 | 6 |  |
| *Lauren classification* | | | | |
| intestinal | 34 | 14 | 20 | 0.351 |
| diffuse | 27 | 8 | 19 |  |
| *Invasive depth* | | | | |
| T1 + T2 | 11 | 5 | 6 | 0.504^a^ |
| T3+ T4 | 50 | 17 | 33 |  |
| *Lymph node metastasis* | | | | |
| presence | 47 | 17 | 30 | 0.975 |
| absence | 14 | 5 | 9 |  |
| *TNM stage* |  |  |  |  |
| I+ II | 21 | 10 | 11 | 0.173 |
| III+ IV | 40 | 12 | 28 |  |
| *ALDH1A1 expression* |  |  |  |  |
| Negative | 32 | 10 | 22 | 0.411 |
| Positive | 29 | 12 | 17 |  |

^a.^ Fisher’s exact test
